# The embarking of COVID-19 and the perishable products’ value chain in Ethiopia

**DOI:** 10.1186/s13731-022-00224-5

**Published:** 2022-03-12

**Authors:** Nasir Ababulgu, Nugusa Abajobir, Hika Wana

**Affiliations:** 1 Department of Agribusiness and Value Chain Management, Faculty of Resource Management and Economics, Wollega University, Shambu Campus, Shambu, Ethiopia; 2 Department of Agricultural Economics, Faculty of Resource Management and Economics, Wollega University, Shambu Campus, Shambu, Ethiopia

**Keywords:** COVID-19, Value chain, Perishable products, Women entrepreneurs, Ethiopia

## Abstract

The aim of this paper is analyzing the impact of COVID-19 on the perishable products’ value chain in Ethiopia. As a methodology, both data sources and types: primary and secondary, qualitative and quantitative, were used to achieve the objective of the study under consideration. The primary data sources used in this work is mainly phone survey, expert opinions and judgments based on real situation observation, and that of secondary data were collected through review of materials published on lessons learned from previous pandemics by different reputable sources. Therefore, this work is based on systematically reviewing and retrieving secondary sources through Google search, library plus harvesting and word type searching. The findings of the study revealed that the COVID-19 pandemic cut the full functioning of the value and supply chain of perishable products due to social distance restrictions imposed by the government, fear of the disease, cutoff transportation and even lock-down of market centers. This led to price changes, gross domestic product loss, the start-up of agro-industrial parks was delayed, reduced export and more women become out of work due to their high participation in perishable products’ value chain. To mention, Ethiopia has lost about $25 million—almost 10% of annual revenue—just over $10 million within the horticultural sector and around 50,000 workers lose their jobs—mostly female labourers. Based on the results, the authors forwarded the collective engagement of the concerned bodies to reduce the negative impacts of COVID-19 on perishable products by using the possible mechanism.

## Introduction

The novel Coronavirus disease (COVID-19) outbreak is a global health event caused by the Severe Acute Respiratory Syndrome Coronavirus-2-SARS-CoV-2 (Ilesanmi & Afolabi, 2020). The COVID-19 pandemic triggered a global economic recession which has resulted in a dramatic loss of livelihoods and income on a global scale (World Bank, 2020). According to food and agricultural organization, the global markets in staple grains remain robust for now; following good harvests in 2019, stocks of most staple foods are adequate. Yet the vast majority of the world’s population takes its food from local markets, and food security and nutrition remain highly susceptible to disruption.^1^ The COVID-19 pandemic highlights the existing gaps in the Nigerian agricultural system, food supply chain, and weakness in absorbing shocks resulting from disease outbreaks. To avert the effects of the COVID-19 pandemic on food losses in the agricultural value chain in Nigeria, much precedence should be placed on adequate stakeholder engagement (Funmilayo et al., 2021).

Measures to control or mitigate COVID-19 outbreaks are already affecting global food supply chains. Border restrictions and lockdowns are, for example, slowing harvests in some parts of the world, leaving millions of seasonal workers without livelihoods, while also constraining transport of food to markets. Meat processing plants and food markets are being forced to close in many locations due to serious COVID-19 outbreaks among workers. Farmers have been burying perishable produce or dumping milk as a result of supply chain disruption and falling consumer demand. As a result, many people in urban centres now struggle to access fresh fruits and vegetables, dairy, meat and fish. Actors in all parts of the food system are impacted by this pandemic. Deep global economic shocks caused by COVID-19 will impact the cash flow and financial liquidity of producers, small and medium agri-businesses to financial institutions, due to inhibited production capacity, limited market access, loss of remittances, lack of employment, and unexpected medical costs (UN, 2020).

Canadian fruit and vegetable markets were significantly impacted by the spread of the novel coronavirus (and COVID-19 disease), beginning in March, 2020. Due to the closure of restaurants, bars, and schools, produce growers and distributors were forced to shift supplies almost entirely from the foodservice to the retail channel.^2^ The study findings from Zimbabwe revealed that extended services and food supply were adversely affected by the outbreak of COVID-19. It was universal among the key informants that travel restrictions reduced farmer-extension worker physical interaction and farmer trainings. This will consequently hamper productivity and increase proneness to hunger as food supply reduces. People need to comply with COVID-19 regulations to flatten the COVID-19 transmission curve (Muvhuringi et al., 2021).

In Italy, fresh and perishable products, whose production or harvest took place during the first wave of COVID-19, have suffered price level effects, while storable products have not registered significant impacts. This phenomenon is mainly due to the vulnerability of the harvest and production phases, which affected fresh and perishable products supply, and to the resilience of transports and logistics, which instead ensured the supply of storable products to the final consumer (Coluccia et al., 2021). In Bangladesh, the COVID-19 pandemic is likely to have substantial effects on the livelihood of people, but smallholder vegetables growers will be even more affected because of the perishability nature of the product. Lockdown has impeded vegetable farmers’ access to markets, thus limiting their productive and sales capacities. The price of yield has dropped by more than half resulting in huge loss for vegetable growers (Alam and Khatun, 2021). With similar fashion, the vulnerabilities in agricultural supply chains and depleted workforces caused by the COVID-19 crisis have hurt farming of all scales and forms in India. Most affected have been dairy farming, floriculture, fruit production, fisheries, and poultry farming.^3^

Moreover, the pandemic came at a time when food security and our food systems were already under strain. Conflict, natural disaster, climate change, and the arrival of pests and plagues on a transcontinental scale preceded COVID-19 and were already undermining food security in many contexts. For example, in East Africa, people are facing a “triple menace” of mutually exacerbating disasters, as ongoing heavy rain hampers attempts to deal with swarms of locusts in the midst of the COVID-19 outbreak^4^. Meanwhile, the worst locust crisis in decades threatens crops heading into the harvest period.^5^ As countries continue to roll out sizable relief and stimulus packages, the needs of food system actors deserve focused attention. Targeted measures to alleviate liquidity constraints on vulnerable firms and households can help facilitate continued production and people’s access to adequate food and nutrition. High-value commodities, like fruits and vegetables, meat, fish and dairy, while readily available for now, tend to be more vulnerable to logistical problems,^6^ because their production is labour intensive and the products are highly perishable.

Ethiopia passed through a violent conflict following major political conflicts which resulted into domestic political shifts. In addition to this, Large-scale displacement, killings, and destruction of property have further aggravated the impact of pre-existing and cyclical climate-related crises across the country. The passed, 2019 and 2020 also brought additional humanitarian crises in the form of a desert locust infestation, Ethiopia’s worst in 25 years, and the global Corona Virus Disease 2019 (COVID-19) pandemic, which counted 119,951 cases in Ethiopia as of 20 December 5 and has led to lockdowns and transport bans in many regions that restrict access to markets. The recent unrest in the northern regions of Tigray has brought large-scale displacement with approximately 41,000 individuals fleeing into Sudan as of 25 November. These events have severely affected the availability of staple market commodities, and sharply escalated humanitarian needs for vulnerable populations (Food and Agriculture Organization, 2020). In a recent survey, Seneshaw Tamru, Kalle Hirvonen, and Bart Minten tried to assess immediate effects on the vegetable value chain in Ethiopia. Their findings suggest that urban consumption of fruits and vegetables is declining, that trade is affected by travel bans and reduced competition, and that farmers face lower prices and reduced access to inputs—Johan Swinnen, series co-editor and International Food Policy Research Institute (IFPRI) Director General (IFPRI, 2020).

In the country, the wholesale market for fruits and vegetables was relocated from crowded quarters to an open space to facilitate social distancing between customers and traders in the country. While these actions were expected to slow the spread of the virus, they may have had substantial unintended effects on the functioning of food value chains. While these actions are expected to slow the spread of the disease, they are likely to have substantial effects on food value chains,^7^ and thus on the livelihoods of farmers and other workers, and on consumption. This implies, many actors involved as a direct actor and supporter inside and outside of the boundary from producer up to final destination are highly affected due to COVID-19 pandemic. Many researches have been conducted on the impacts of COVID-19 on food security, recent macroeconomics, and many other cereal products, but very few research were conducted on perishable products and this work was expected to contribute and fill the gap of the impacts of COVID-19 on perishable products and its value chain in Ethiopia.

In this paper, we tried to innovate and close the gap of other researches on perishable food products’ value chain with the inclusion of the critical variables: prices, marketing, consumption, women, labor and other several issues related social protection system in the value and supply chain network economic activities. This work extends its area of concern to foreign direct investment especially horticultural crops due to its nature of perishability and actors in the overall value chain. Such an integration and introduction can provide valuable insights for the management and analysis of perishable food value and supply chains during the pandemic. This work also adds to our and readers understanding of complex phenomena associated with dynamics of disasters and impacts of the pandemic on smallholders’ livelihoods in Ethiopia. The contributions in this paper set the stage for research on other perishable product value chains in which they are subject to disruptions as we are witnessed during the COVID-19 pandemic.

## Review of literature

The pandemic's impact on food availability and prices depends on what happens to the demand and supply of foods. At the beginning of the pandemic, many experts feared that the crisis would lead to food price increases (e.g., Reardon, et al., 2020). So far, global staple food prices have been remarkably stable, most likely due to good harvests in the previous season and sufficient global storage. In confined spaces such as packing plants for fruits and vegetables or meat processing facilities, necessary social distancing measures may reduce the efficiency of operations and there is a need to ensure adequate protections for employees. Many firms have also reported high rates of worker absences; for example, staff availability was reduced by up to 30% in French meat processing facilities in the regions of the country worst hit by COVID-19.^8^

Perishable foods chains, such as fruits and vegetables, meat, milk or dairy products have been particularly impacted: they often require many people to work in close proximity to cultivate, harvest and process. Those fresh products are highly nutritious and essential to healthy diets. Informal markets had to close due to containment measures, reflecting a “formality bias” in policies, which favored supermarkets because they were perceived as safer. Yet informal markets play a central role in ensuring access to diversified and nutritious foods. This further undermined access to local healthy diets. COVID-19 clusters have been found in meat processing plants in various countries. Employees often work in close proximity to each other, making it more difficult to respect physical distancing requirements. In some cases, workers also live together in overcrowded conditions, which further facilitates the spread of the virus.^9^ Meat processing appears to be more sensitive than other types of food processing in part because of the labor-intensive nature of operations. By contrast, grain handling and processing is highly automated and less labor intensive, and has not experienced the same disruptions as the meat processing sector.

In China, where the production, distribution, and sales of vegetables and other agricultural products were adversely affected by the pandemic. In addition, a disruption of the seed supply chain, agrochemicals and fertilizer importation resulted, thus, reducing viable seeds, and limiting the yield of agricultural produce (Hai-ying, et al., 2021). In Nigeria, the markets for perishable food items were unfavorably affected by workplace closure at the processing and packaging departments of food companies. The transportation of these perishable produce to the market were hindered as a result of travel restrictions put in place, and this resulted in an increase wastage of food items. The increasing rate of COVID-19 cases also necessitated shift duties, thus reducing available manpower to ascertain food safety and quality (FAO 2020).

High levels of animal protein deficiency have been reported in Nigeria. The livestock production rapidly bridges this deficit. This is due to the limited period of growth and regeneration, as well as the presence of large protein portions. More often, livestock bred at the household level play a crucial role in alleviating poverty, enhancing income generation, and assuring food security. The COVID-19 pandemic has, however, resulted in a reduction of livestock production both in small and large scales (Oyetoro, et al., 2020). This event primarily stems from the reduced money in circulation during the COVID-19 pandemic, and reduced earning among many individuals. Thus, the purchase of livestock feeds and drugs seemed unrealistic. For this cause, household savings in the pre-COVID-19 period in Nigeria was expended on basic food items which could assure of survival. For many households, although food items were available at the market, nutrient-rich foods including vegetables were not accessible, and thus could not be utilized for body growth and development.

For instance, in Bunia (Ituri), a price surge affected all staple food (cassava, corn flour-local or imported, palm oil, beans) immediately after the announce of anti-COVID measures. This price surge attained up to 50% for beans, and 100% for local corn. Prices have since decreased, yet they remain higher than the same period last year. Only cassava seems to be back to normal. In Tshikapa (Kasaï), there is a similar trend: the price of vegetable oil (mostly imported from Angola) has doubled. The price of imported rice has increased of 21%. Most local crops (cassava, beans, local corn flour) have known a slight decrease in May–June, which corresponds to harvest months, but prices are re-increasing fast since and are higher than last year.

In recent years, Ethiopia has been battling with double-digit inflation rates. Just before the pandemic began, annual food inflation was 21% (New Business Ethiopia 2020). The latest inflation estimates from July 2020 puts year-on-year food inflation at 24%, suggesting that food prices have risen somewhat faster during the pandemic than before (Addis Fortune 2020). Meanwhile, IFPRI's vegetable value chain survey in the main value chain connecting farmers in East Shewa zone in Oromia to consumers in Addis Ababa suggests that price changes vary enormously across crops. For example, in Addis Ababa, retail prices of tomato and onion increased by 33% and 20%, while the prices of green pepper and cabbage went down by 13 and 12%, respectively (Hirvonen, et al., 2020). For some crops (e.g., onion), transportation disruptions and border closures meant a serious decline in supply as imports from regional states or neighboring countries, e.g., from Sudan in the case of onions, came to halt. For other crops, e.g., green pepper, there was over-supply as exporting to neighboring regional states became more difficult. Lower incomes due to COVID-19 disruptions and misperceptions that the virus is transmitted through certain foods contributed to lower demand, especially in urban areas (Hirvonen et al., 2021).

## Methodology

### Data sources

The primary data sources used in this work is mainly phone survey, expert opinions and judgments based on real situation observation, and that of secondary data were collected through review of materials published on lessons learned from previous pandemics by different reputable sources, such as International Food Policy Research Institute (IFPRI), Food and Agriculture Organization of the United Nations (FAO), International Livestock Research Institute (ILRI), World Bank (WB), United Nations Office for the Coordination of Humanitarian Affairs (OCHA), World Health Organization (WHO) and Ethiopian Economic Association (EEA). Therefore, this work is based on systematically reviewing and retrieving secondary sources, in addition to our observation made on the impacts of COVID-19 on value chain of perishable products, actors of the value chain, its marketing, prices, and consumption, processing and value addition through Google search, library plus harvesting and word type searching.

### Analysis and writing

The above mentioned materials were compiled, analyzed and written systematically based on the scientific write-up requirement of the paper. First, the data`s are organized and searched through searching important concepts related to the issue under consideration. Second, the necessary points related to COVID-19 and value chain of perishable products value chain were identified. Third, they were analyzed and presented accordingly.

## Results and discussion

The results of the study are organized and discussed as follows. In "The impact of COVID-19 on Agricultural Sector focusing on Value" and supply Chain, lobers and actors, value addition, processing, marketing, export, prices of perishable products are clearly presented. We then discussed COVID 19 and the Consumption of Perishable Products, in "Impacts of COVID 19 on the consumption of perishable products". "COVID-19 and Women Entrepreneurs in Ethiopia", deals with women entrepreneur during the era of COVID-19, and finally, conclusions and implications of the findings were made under section seven.

## The impact of COVID-19 on agricultural sector and its value chain

Possible impacts of COVID-19 on the Agricultural sector and its value chain especially on perishable products have many implications to work with different concerned bodies and stakeholders to reduce the negative impacts that are resulted due to the outbreak of the disease. According to^10^COVID-19 has coincided with the beginning of the Belg rainfall season. The emergency could be expected to worsen malnutrition with preventive social distancing limiting access to health facilities and markets, coming at a time of deteriorating food security. On-going emergencies will complicate underlying seasonal challenges that vulnerable communities face in accessing adequate food and income. As a result, the most vulnerable products affected severely before being marketed though demands are many but unable to obtain the marketing access due to the restrictions made to save the life of the marketers and the overall society from the COVID-19. The embarking of the pandemic laid down a lot of losses on agriculture, food, marketing and products’ value chain in the following ways:

### Productivity in high potential areas may be compromised

The output of high agricultural production areas in western Ethiopia which depends on inputs and labour could be negatively affected by COVID-19 in several ways: (i) travel and movement restrictions will suppress the supply market, thus, negatively impacting access to required agricultural inputs like seeds, agrochemicals and fertilizers; and (ii) the spreading/fear of COVID-19 infection among the population will decrease the supply/availability of the labour force for traditionally labour intensive farming systems.

### Disruptions to income earning opportunities during important festivities

Through demand and supply side shocks, the crisis may disrupt food systems, thus, threatening jobs in each segment of the system. Labour in agriculture is becoming scarce, mainly in labour-intense value chains like horticulture, affecting rural workers and employment of internal seasonal migrants. This could be driven by faltering SMEs in agriculture and other sectors which lack the capital to overcome a short-term drop in cash flow or disrupted access to markets for their produce, in addition to restrictions on public travel and gatherings. As a result, household incomes are likely to be affected negatively by reduced employment in rural areas thus impacting on a diverse range of human rights concerns including right to adequate standard of living; rights to food, water, housing and access to basic goods and services necessary for the continuous improvement of living conditions.^11^ In addition, some important religious festivals for orthodox Christian and Muslim faiths will be severely affected, with implications on household occasional incomes and expenditure patterns. This is likely to be exacerbated by a reduction of remittances, due to the global economic effects of the crisis and increased repatriation of Ethiopia workers from some Gulf countries.

### The functioning of agro-industrial parks (AIP) will be compromised

The agro-industrial parks are meant to be the backbone of modernization in the agriculture sector by improving production and productivity (quantity and quality) and commercializing and industrializing strategic agro-commodities through improved market linkages. Travel restrictions have already destabilized the flow of agricultural commodities to the parks thereby affecting the entire food value chain including processing, transportation and distribution of food crops.

### Production, productivity and market access segment can be impacted by poor availability of inputs

(fertilizers, seeds, animal feed, ph11ytos, veterinary products and services and so on). Most rural Ethiopian producers are operating at very low productivity and are highly vulnerable to shocks. The impact on production and productivity will be limited if imported inputs are delivered for this season and distributed to producers. Impact would be significant if the inputs are not yet distributed or not yet imported. Research data^12^ show that agricultural production might drop by 30% if producers revert to the extensive production system for cash crops: Along with the effect of lower productivity, rural incomes could also suffer from limited opportunities to sell goods. This will lead to price increases, aggravating food insecurity, malnutrition and, ultimately, vulnerability of the population to poverty hence aggravating already existing inequalities and social deprivation among the most marginalised and vulnerable groups.

### Access to production capital includes, among others, access to finance

Is to pay for the cost of inputs, labour, mechanization services, transport services, fees related to irrigation systems and so forth. Normally, a part of the cost of such charges is covered through financing from rural micro finance institutions established in different regions, zones and even woredas. Depending on the intensity of the crisis, this could be comprised significantly, opening the way for informal lending systems and possible exploitation of an already poor population. This could put women and female youth in a disadvantageous position as their access to financial service and related services in rural settings may be more limited, coupled with ongoing restrictions in movement and the burden of household work and care giving responsibilities.

### Pastoralism and livestock sector

People in Ethiopia’s lowlands are largely pastoralists and agro-pastoralists. Livestock products represent an important source of food intake for these communities, as well as exports and food sources for urban people. The livestock sector under intensive production system (fattening centres) may be knocked out following feed shortages due to restrictions on movement and reduction in agro-industrial production. The main export market during the Ramadan season in May and Hajj season in August will be lost and revenues from live animal exports (24.3 million kgs exported for a value of USD 45.8 million). A decrease in live animal commercialization in the country will have severe repercussions on meat and meat products (17.7 million kg valued at USD 88.6 million), and textile industry including leather and leather products (5.6 million kg valued at USD 117.4 million.

### Fruits and vegetable production

A recent survey by IFPRI showed that labour intensive and highly perishable horticulture value chains have already been impacted by decreased domestic trade and consumption of vegetables, despite the Orthodox fasting season, shortage of and increased input prices, increased farm losses, travel bans that impacted the volume and frequency of truck movements, decreased purchases for restaurants and eateries, and misconceptions related to fresh food contamination.^13^ This subsector will be severely impacted, in both export and local markets, due to the perishability of commodities and the disrupted domestic distribution system. Without facilities to export, prices will drop very quickly if the harvesting season arrives before the end of the pandemic. Other key export commodities will also be impacted by the ban of international flights. These commodities include pulse (462.8 million kgs valued at USD 272.3 million), oilseeds (260.9 million kgs valued at USD 387.8 million) and coffee (230.9 million kgs valued at USD 764.1 million).

### Food availability

Movement of food commodities from surplus producing to deficit areas will be constrained through panic purchases, transporters’ fear of travel and farmers withholding food for their own households. Ethiopia is also approaching the main hunger season for a significant proportion of the population, from June to September, during which food prices normally rise as stocks from the main Meher harvest get exhausted. The performance of the Belg Season (February to May rains) is important for Belg crop producers and for livestock production in all pastoral as well as many crop-dependent areas. Despite a later start, Belg crop producers are receiving near average rains so far, and rains also started in pastoral areas in April. The desert locust invasion, however, is threatening the Belg season.

### Food access

The outbreak of COVID-19 will negatively impact physical access to food, especially in areas where households are already facing deficits due to other factors, for example, below normal rains and displacement. Inadequate supplies of food in markets will reduce diversity of food items consumed by households. Incomes for households in the informal sector will be reduced during the COVID-19 period, worsening the food security situation. Reduction in remittances from abroad will also affect both rural and urban households. Food prices are already unseasonably high; therefore, further increases will worsen the food security situation. The latest data show that food price inflation had already reached 26.9% in March 2020, the highest level since 2012. This could have an impact on right to food by vulnerable and marginalised groups such as older persons, homeless female headed households and compromise their ability to meet required dietary needs. Concerted effort should be made to ensure physical and economic access to food by all; targeted action should be made to support vulnerable and food insecure households and groups.

### Food utilization

Households have additional needs to prepare food commodities for consumption, including the cost of the fuel and access to water. Some of the food items in the basket will also require pre-processing, including milling, which will impose additional costs for the poor and vulnerable faced with loss of livelihoods, income and assets. Source.^14^

### The impact of COVID-19 on value chain of perishable products

COVID-19 laid down different impacts on perishable value chains, the share of actors along the chain especially producers, its prices, processing and value addition which result in the loss at producer level, actors gains, reduced export and the overall economy. As the result of the outbreak of the pandemic, producers are not allowed to supply their perishable produce to the market as before due to social distance restrictions imposed by the government, cutoff transportation and even lock down of market centers. Though producers tried to take some measures to save themselves from these losses through cash position (delayed payments to suppliers, efforts to collect customer payments and delayed investments) to ensure continuity in the short term, the negative impacts of COVID-19 on their produce and returns where highly visible and significant enough. The central market of perishable products in Addis Ababa, the capital city of the country was affected which in turn affect the whole value chain actors putting the producers in bankrupt particularly. Aday and Aday (2020) reported that in a study in the USA, 70% of consumers reduced the frequency of shopping. This resulted in those farmers into perishable agricultural produce production making losses as the market was performing below the expected due to prohibition of common market place.

Relying on the work of researchers (Seneshaw, et al., 2020)*,* the COVID-19 pandemic is beginning to disrupt food value chains in Ethiopia and elsewhere, impacting the livelihoods of farmers and the diets of rural and urban households. These effects are likely to hit the poorest and most vulnerable farmers and consumers the hardest, but they are not yet well understood. More evidence is needed to guide the government and other organizations in devising responses. While not representative of the whole value chain, their interviews with vegetable production stakeholders have a number of potential policy implications. First, urban demand for fruits and vegetables—high-value, nutritionally rich foods—is declining. It is possible that this is driven by misinformation regarding the risk of contracting COVID-19 from produce. If so, there is a need for widespread and effective information campaigns. Second, trade is affected by travel bans, as well as reduced competition, because traders are less willing to travel to production areas. Making sure that travel bans do not negatively affect food trade is paramount. To reduce the need for travel, enhanced trading through smartphones, virtual purchasing, and e-payments could be considered so that only truck drivers who pick up loads would need to travel, and traders and brokers would not need to travel. Third, farmers are apparently hit in two ways. Producer prices are lower, and input prices are up or inputs are not available. Farmers will thus have less incentive to produce these crops, likely leading to lower yields and production in the near future. To avoid further disruptions to the food supply, ensuring the availability of agricultural inputs to farmers at low prices and assuring incentives for production should be a priority for the government in the next few months.

### Demand for high-value perishables is shrinking

As people lose jobs, firms cut production, the government diverts investment to relief response, and the overall demand for perishable food products falls. Demand for more expensive and perishable products that are often consumed in hotels and restaurants has fallen sharply (e.g. milk, butter and meat), as has the demand for products that are believed to increase susceptibility to COVID-19 (e.g. cabbage and tomato) (RCA, 2020). This, in turn, leads to even more job losses along these value chains, Income from remittances has declined, because the diaspora residing in other countries face income and job losses due to the measures taken and the national poverty rate rose by 9% during the lockdown period; an additional 10 million people were recorded as temporarily living below the poverty line—1.90 United States dollars (USD) a day—between March and May 2020.^15^

According to different evidence, the export-oriented fruit sector in Ivory Coast and horticulture sector in Ghana are of considerable importance for the economy of the countries. In Rwanda, the horticulture sector has ambitions and potential to grow. Yet in all three countries the COVID-19 crisis had great impact on the sector. In Ghana, restrictions in mobility led to a drop in exports of fruits and vegetables, alongside reduced demand in markets, such as schools and hotels. Income of farmers and out-growers decreased sharply. This threatens livelihoods and weakens resilience to future shocks. Due to a lower income, combined with a general lack of access to finance, farmers are hampered in purchasing inputs for the upcoming growing seasons. Ivory Coast was hit hard too and faces similar issues as Ghana. Closure of the main French markets during the peak of the COVID-19 pandemic stopped export of mangoes and other fruit. The result was a decrease in household income and operational capital, threatening livelihoods. As in Ghana, lack of income and limited purchasing power for inputs might well threaten the upcoming production seasons.^16^

### Value addition and processing of perishable products

In the country, since the outbreak of COVID-19, the government set policies to reduce the number of labor force working in the organizations, bans traveling and reduce volume of transport per car, forced to lock down some processing plants or industry. Few industries, processing the vegetable and fruit products were closed and as a result the demand for the fruit and vegetable value added products decreased, employees leave their daily activities and forced to stay at home. As a result, the demand of the previously processed vegetable and fruits becomes highly increased since the lock down of the industries.

GDP loss in food processing was 12% in the first weeks of the outbreak. The start-up of agro-industrial parks will be delayed (Rapid Country Assessment, 2020). A monitoring survey of 3107 households in Ethiopia, conducted in two rounds by the World Bank^17^ indicated that labour and distribution costs are higher; urban areas, where shops are running out of stock owing to delays in deliveries, have been most affected (11% of the surveyed households), while rural areas have been impacted by markets not operating (9% of the surveyed households).

### Supply chain of perishable products and COVID-19

COVID-19 has affected all spheres of the global supply chain including distribution and packaging, as well as sourcing of raw materials**.** Reduced travel reduces the mobility of goods and people which directly affects the supply chain. In Ethiopia, the dominant producers of the perishable products are small holder farmer, rural residents. On the first outbreak of the virus, the gov`t restricts social distance, transportation volume and cross border traveling. As a result, producers and other middle actors restrict themselves from supplying the products to the final market. This made supply chain not to fully function. Relying on FAO (2020), the closure of many informal markets in the urban and peri-urban areas to avoid crowding has disrupted food supply systems, especially for fresh produce, such as meat, eggs and milk. In view of this, shifts in consumer demand have been reported. The impact is felt mostly in low-income urban households who rely on these informal food markets. Middle- and higher income families can buy fresh produce from supermarkets and grocery shops. The livelihoods of fisheries and aquaculture actors will also be negatively affected due to decreases in consumer demand, disruptions in markets access and logistical bottlenecks. For pastoralists, there will be the loss of income from selling livestock and products (milk, butter, gee and eggs), resulting in an increased reliance on the environment (charcoal burning) and overall reduction in the purchasing power of households.^18^

#### Marketing and supply chain linkages

Marketing is a common challenge that micro and small start-ups face. This is also suggestive of the difficulties MSEs may face in crisis situations like that precipitated by COVID-19. The same survey indicated that 61% of MSEs do not have a sales outlet and only 5% have more than one sales outlet. The implication is that most enterprises sell directly to final consumers, a feature that adds to their vulnerability as consumer demand has fallen already and consumers’ ability or willingness to go to markets as per normal practice has been inhibited by restrictive measures as well as personal concerns. Marketing capacity also varies according to size of the firm and location (region vs Addis). The larger the firm size, the more diversified its market base; as expected, businesses in Addis have a more diversified customer base and range of sales outlets than those in the regions. In terms of inputs/raw material supply, the majority of MSEs (about 87%) have domestic private enterprises as main sources of productive inputs, suggesting a cascade effect as these sources themselves face challenges in sustaining themselves in business in the face of a large-scale crisis. Though about 9% of the MSEs have State Owned Enterprises (SOEs) as their sources of productive inputs, other sources—such as foreign investors, non-commercial entities and direct imports—were utilized by less than 4% of enterprises. Only few enterprises (1.5%) are dependent on imported raw materials (UNE, 2020).

The significance of COVID-19 for food supply chains thus comes less from its impact on primary production or overall food demand than from its disruptive effects on the complex web of actors connecting farm to fork, and the sudden change in the demand mix. Disruptions in processing, in particular for meat, can “disconnect” supply and demand, creating simultaneous surpluses for producers and shortages for consumers, while for some specific products demand has also decreased, leading to a temporary oversupply (e.g. potatoes for French fries, or milk for cheese).^19^ At the same time, shoppers sometimes experienced empty shelves in supermarkets during the early days of COVID-19, as food supply chains adjusted to the sudden demand surge.

The risk to food security currently does not come from disruptions along supply chains, but rather from the devastating effects of COVID-19 on jobs and livelihoods. Especially in developing countries, where social safety nets are less well-developed, COVID-19 may lead to a severe increase in poverty and hunger.^20^

Therefore, the implications from the above evidence implies, the COVID-19 pandemic has placed unprecedented stresses on food supply chains, with bottlenecks in farm labour, processing, transport and logistics, as well as momentous shifts in demand. Most of these disruptions are a result of policies adopted to contain the spread of the virus. Food supply chains have demonstrated a remarkable resilience in the face of these stresses. Grocery store shelves have been replenished over time, as stockpiling behaviour disappeared and as supply chains responded to increased demand. Long lines at borders shrank quickly in response to policies to alleviate unnecessary restrictions. While the impacts of COVID-19 are still unfolding, experience so far shows the importance of an open and predictable international trade environment to ensure food can move to where it is needed (OECD, 2020).

### COVID-19 and foreign direct investment of horticultural crops

In Ethiopia, crops like tomatoes and onions are consumed in almost all the three daily meals, are eaten either raw or cooked during breakfasts, lunches and suppers. Therefore, the crops have a higher daily demand compared to non-horticultural crops, such as maize, sorghum hence are produced oftentimes. However, the horticultural crops are highly perishable so reliable markets are an essential if commercially produced. A delay in marketing after harvesting will result to a rapid loss of saleable quality and quantity. If farmers produce more than what the available market can absorb, there are increased chances of post-harvest losses especially if there are poor or no post-harvest handling facilities. Therefore, commercial production levels of horticultural crops have to be balanced with the available markets. Marketing of horticultural products is dynamic so limited knowledge about the markets will result to huge losses. Regrettably, quantities of horticulture produce losses from spoilage (15%) and market glut (60%) is significant especially during the peak production periods. The produce losses are caused also by the limited access to higher value urban markets by the smallholder horticulture producers. The sudden outbreak of COVID-19 led to an unexpected severe imbalance between the production levels and market availability (Parwada, 2020).

Horticultural crops are one of the crops which attract the foreign direct investment to the country. Hence, as the pandemic outbreak, the government stops integration with the world which resulted into the fall of foreign direct investment to the country, Ethiopia. COVID-19 has had a broad-based negative impact on trade and foreign direct investment, with considerable declines in global exports and imports in a range of industries. The impact is likely to worsen in the near future as countries move progressively through phases of the pandemic, with world trade expected to contract by between 13 and 32 % in 2020 (with all regions experiencing double digit declines in trade volumes), and foreign direct investment expected to fall by 30–40% in 2020–21. Initial disruptions in Global Supply Chain (GSC) started on the supply side with factory closures in China, instituted to slow the spread of COVID-19. This situation eventually resulted in shortages of parts and equipment to downstream industries—most notably the automotive, chemicals, computer equipment, garments and textiles, machinery, metal and metal products industries, and those relating to precision instruments (United Nations Conference on Trade and Development, 2020).

The sequential effects of these shortages reverberated in many other countries, causing some enterprises to slow production or cease operations altogether. Furthermore, the negative labour supply shock due to national lockdowns and the restrictions on cross border movements of people are contributing to serious supply disruptions for agricultural goods, especially in developing and emerging economies, and many industrial goods. A significant share of trade in some developing economies includes informal cross-border trade; for example, it is estimated that 30–40% of cross-border trade in Africa is informal (UNCTAD, 2020). The subsequent impact on global trade in intermediate manufacturing goods is expected to be particularly acute in 2020, starting with a sharp decline in exports from China, which contributes roughly 20%.

The Ethiopian Horticulture Producer-Exporters Association (EHPEA) has indicated that Ethiopia has lost about $25 million—almost 10% of annual revenue—just over $10 million within the horticultural sector and around 50,000 workers could lose their jobs (mostly female labourers) (Royal Flora Holland, 2020). However, EHPEA is lobbying hard for companies to provide leave for employees rather than face redundancies.^21^ It is difficult to obtain more specific information on effects, though it is known that producers tend to specialise in the Dutch auction house sales—where prices have collapsed, with 85% of turnover gone.^22^ The study by Parwada (2020) tells us the lockdown due to COVID-19 outbreak resulted to low sales and most farmers were unprepared for any crisis during the marketing stage of their production. The research contributed to an understanding of how a crisis situation influence marketing of horticultural produce and raises awareness regarding post-harvest losses.

### The impact of COVID-19 on value chain actors of perishable products

#### Impacts at the farm level: disruptions in input supply, labor availability and extension services

The COVID-19 pandemic coincided with the start of the long rains in Ethiopia and is the topmost season for labor-intensive staple food and vegetable production across the region. Since vegetable products are labor intensive, human movement restriction since the outbreak of the COVID-19 resulted into the shortage of man power especially for perishable and high value products. The countries are also reporting COVID-19-related disruptions to access to agricultural inputs (seed, fertilizer, veterinary inputs, fish fingerlings and feed), which will likely drive a reduction in crop yields. Following the outbreak of the COVID-19 measures imposed reduces farmer’s access to extension service, credit access, market access, transportation access and access to advisory services by Research center and universities during the critical times. According to FAO^23^ identification, the impacts of COVID-19 on disruptions in input supply, labour availability and extension service and the agro-food value chain interruptions in logistics, processing and market access.

As the outbreaks of the virus, the price of vegetable produce becomes declined since the government bans the movements of the traders; fewer traders are traveling to rural areas, the social distancing policy, and fear of infection. According to (IFPRI, 2020) in addition to the decreased urban demand and oversupply, producer prices are rapidly declining. For example, a quintal (100 kg) of head cabbage that sold for about 300 birr ($9) about 2 weeks earlier sold for only 100 birr ($3) at the end of March. Similarly, onions that sold for 15–17 birr ($0.50) per kg about 2 weeks earlier were selling for about 9–10 birr ($0.30) per kg at the end of March, a 40% decline. Due to the decline of the demands of the products farmers leave vegetables and fruits on the fields because of lack of buyers. There is shortage of farm inputs and their prices are increasing in the countries. Prices of important inputs crucial to vegetable production including fungicides, insecticides, herbicides, fertilizers, and improved seeds are increasing due to shortages. These seem to be linked to land border closings, which have blocked (sometimes illegal) imports from neighboring countries, and to reduced imports from China.

#### Causal laborers and non-farming households during the COVID-19

A nationwide ban measure, together with the minus of laborers in the organization and reduced access to markets, give rise to job loses, as a result low income societies which reduce their purchasing power, and push the poor as they follow negative copying strategy and broadening the poverty gap in the country. Related to the agricultural sector, these include casual labourers supporting on-farm planting or harvesting activities (including migrant labourers), transport operators, petty traders, market vendors, and village-based loan and credit operators. The closure of local and farmers’ markets is limiting access to nutritious foods, such as fresh fruits and vegetables for the urban poor. Job losses, combined with a drop in remittances, will limit households’ ability to afford healthy diets and basic needs in the countries.

According to RCA,^24^ prediction there is severe job losses which will continue in the coming months, both in the formal and informal sector. Currently, casual laborers, of which the majorities are people under 35, will experience the heaviest job losses. A monitoring survey of 3107 households, conducted by the World Bank in April and June 2020, indicates that 38% of casual labourers have lost income and/or their job. This is mostly in the service sector, but also in agriculture (e.g. street vendors, food processors). Another major loss of income is expected among (young) casual labourers who cannot work or go to their workplace due to restrictions in movement. The majority of casual labourers in the agriculture sector are young male students, young landless farmers, and young women (the latter mostly in export sectors).

### Perishable products’ marketing during the pandemic, COVID-19

#### Marketing of perishable products

Demand for high-value perishables is shrinking ass people lose jobs, firms cut production, the government diverts investment to relief response, and the overall demand for perishable food products falls. Demand for more expensive and perishable products that are often consumed in hotels and restaurants has fallen sharply (Potato, milk, butter and meat), as has the demand for products that are believed to increase susceptibility to COVID-19 (cabbage and tomato). This, in turn, leads to decline of the demands of perishable products along the value chain.

According to Nasir and Hika (2021), a 25–30% drop in exports of goods and services during 2020 is possible: flower exports declined by 80% in Q1 and are now stopped—the plan was to generate USD 450 million from these exports, with USD 225 million realized in the first 6 months of the Ethiopia FY (fiscal year); textile exporters obtained United State Dollar (USD) 100 million over the same period but faced cancelation of orders from the United State of America (USA), Europe, and China; tourism contributed about USD 600 million in 2018/2019, and this will take a very hard hit with the severe restriction of air travel and its possible continuation into the holiday season and beyond; and Ethiopia Airlines, which has already lost USD 550 million in Q1 2020, could see a doubling or trebling of this figure, reducing net income with losses becoming a real possibility—depending on the duration of air travel restrictions and speed of pick-up in passenger traffic.

The export–import gap was already significant in past years. Ethiopia imports five times the value of what it exports. This gap is likely to increase this year as the international demand for export crops is dropping (except for coffee) (Geda, 2020). According to IFPRI,^25^ Value chain agents indicated that their businesses were seriously affected by the COVID-19 pandemic. Most agents reported a decrease in demand, turnover, and clients; increased losses; less competition; higher transport costs; and changes in procurement areas. As a result, prices changed significantly over the period, with increases over a 3-month period up to 64% (tomato) and decreases as high as 67% (green pepper). Changes at the retail level were, however, relatively much smaller, between 19% increases (tomato) and 29% decreases (green pepper). Marketing margins of the perishable products declined. Moreover, increases in transportation costs, which were seen in Ethiopia as well as in other countries during the pandemic (Narayan and Saha 2020), might have been less of a driving factor of overall price changes in these value chains of perishable products. As indicated by Seneshaw, price formation after the COVID-19 is rapidly changed in the short run.

#### Perishable products’ price crisis with COVID-19

**Fig. 1:**
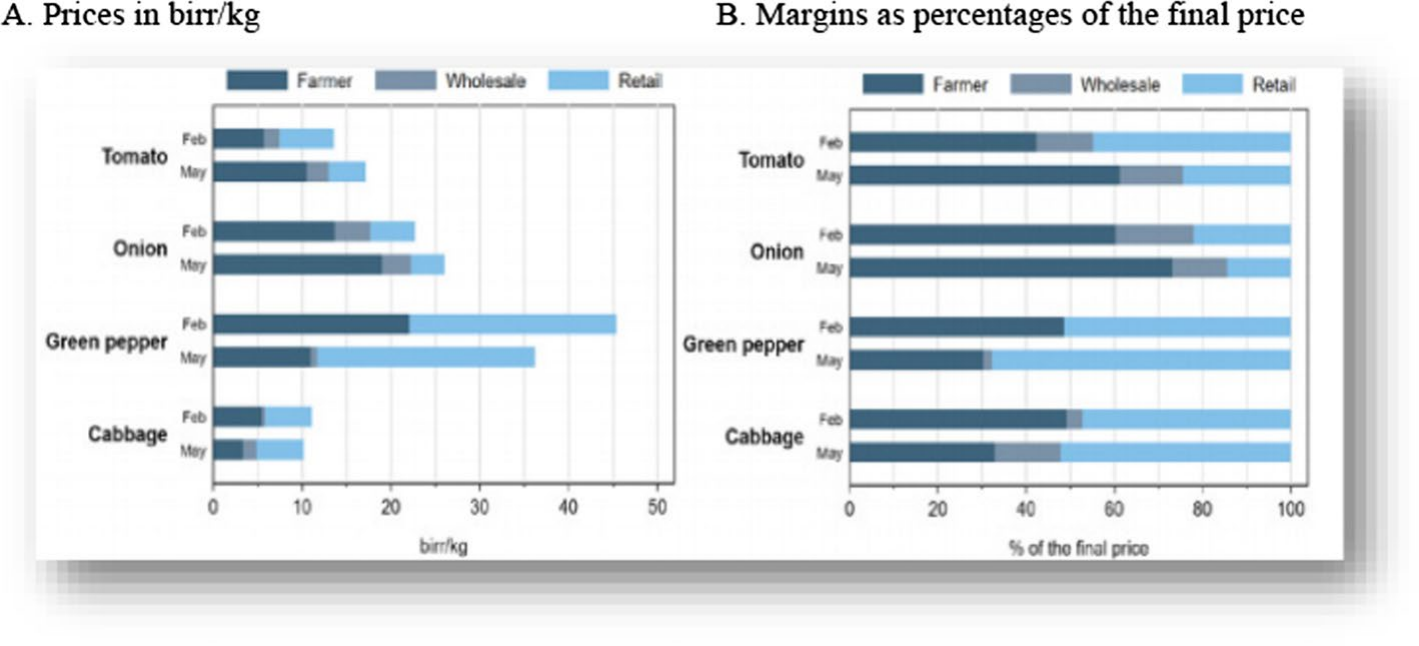
Vegetable price structure before and during the pandemic, by vegetable type. Source: (IFPRI, 2020). **A** Prices in birr/kg. **B** Margins as percentages of the final price

In Ethiopia, the prices of the commodities are highly fluctuated because of the COVID-19 pandemic. As a result, margins obtained at the farmers, wholesalers and retailers level were changing over time. For instance, the prices of tomato, onions, green paper and cabbage show a great change in price in birr/kg and margins as a percentage of the final prices (Fig. 1).

The margin of actors along the value chains started to decline due to limited and irregular transport, its perishability, deflated prices of commodities and limited wholesalers. This is in line with Hervonen et al., (2020) in which they find large, but heterogeneous, price changes for different vegetables with relatively larger changes seen at the farm level, compared to the consumer level, leading to winners and losers among local vegetable farmers due to pandemic-related trade disruptions. They further note that despite substantial hurdles in domestic trade reported by most value chain agents, increases in marketing—and especially transportation—costs have not been the major contributor to overall changes in retail prices. Marketing margins even declined for half of the vegetables studied.

The evidence provided by some researchers suggested that this led to substantial changes in vegetable prices. In particular, they identified large price changes for farmers, but the effects are heterogenous: farmers who faced less competition from other areas (locally or internationally) benefited through higher output prices from the imposed pandemic trade restrictions, while those that could no longer export to other areas in the country lost out. Overall changes in wholesale and retail marketing margins have been relatively less important, despite the reduced turnover, higher losses, and higher transportation costs reported by agricultural traders and retailers. They take this as evidence of notable resilience in the local marketing systems.^26^ The study by Muvhuringi (2021) revealed that COVID-19 has also reduced food access for the urban low resource consumers as staple food prices have increased and these urban poor got their farm produce from the market place, such as Mbare musika in Harare which was shut down in efforts to contain the pandemic.

These emperies have important implications for policy. First, close monitoring of price movements and the factors contributing to those movements is paramount, especially during this crisis period. Changes in consumer prices are often claimed to be linked to predatory behavior among traders, motivating government intervention to curb trading activity, as has already been witnessed during the COVID-19 pandemic (Resnick, 2020; Wegerif, 2020; Gebreamlak, 2020). However, the earlier evidence on such predatory behavior is limited^27^ and the other findings indicated that the price changes during this pandemic have not been driven by large increases in marketing margins. Second, quantitative assessments on the relative importance of different segments in the value chains are useful for setting priorities to reduce farm-retail spreads in order to achieve higher prices for producers and lower prices for consumers. This implies lower importance of transportation costs and the large contribution of urban distribution costs in the final retail prices of vegetables. More emphasis on addressing poor efficiencies in these urban distribution systems is, therefore, called for. Parwada also reported that the highest (> 35%) horticultural surpluses at the markets and spoilages during the lockdown.

## Impacts of COVID-19 on the consumption of perishable products

Vegetable value chains are being affected due to the perishable nature of the products, which cannot be transported effectively due to the restrictions in mobility. Vegetable trade and consumption have also decreased. The COVID-19 pandemic reduces the productivity, consumption and marketing of the perishable products. Following this, the demand for vegetable products are reduced and some especially larger and wealthier traders are taking precautionary measures to avoid exposing themselves to the virus as a result it reduces the vegetable trading activity, the travel bans also reduced the volume and frequency of trucks coming to market center of the countries**.** Finally, many hotels and Restaurants choose less vegetable purchases since the slowdown of the business in the countries. In general, both supply and demand appear to be impacted simultaneously: The lower urban demand that typically leads to a reduction in vegetable prices is balanced by a declining vegetable supply to Addis Ababa.

**Fig. 2:**
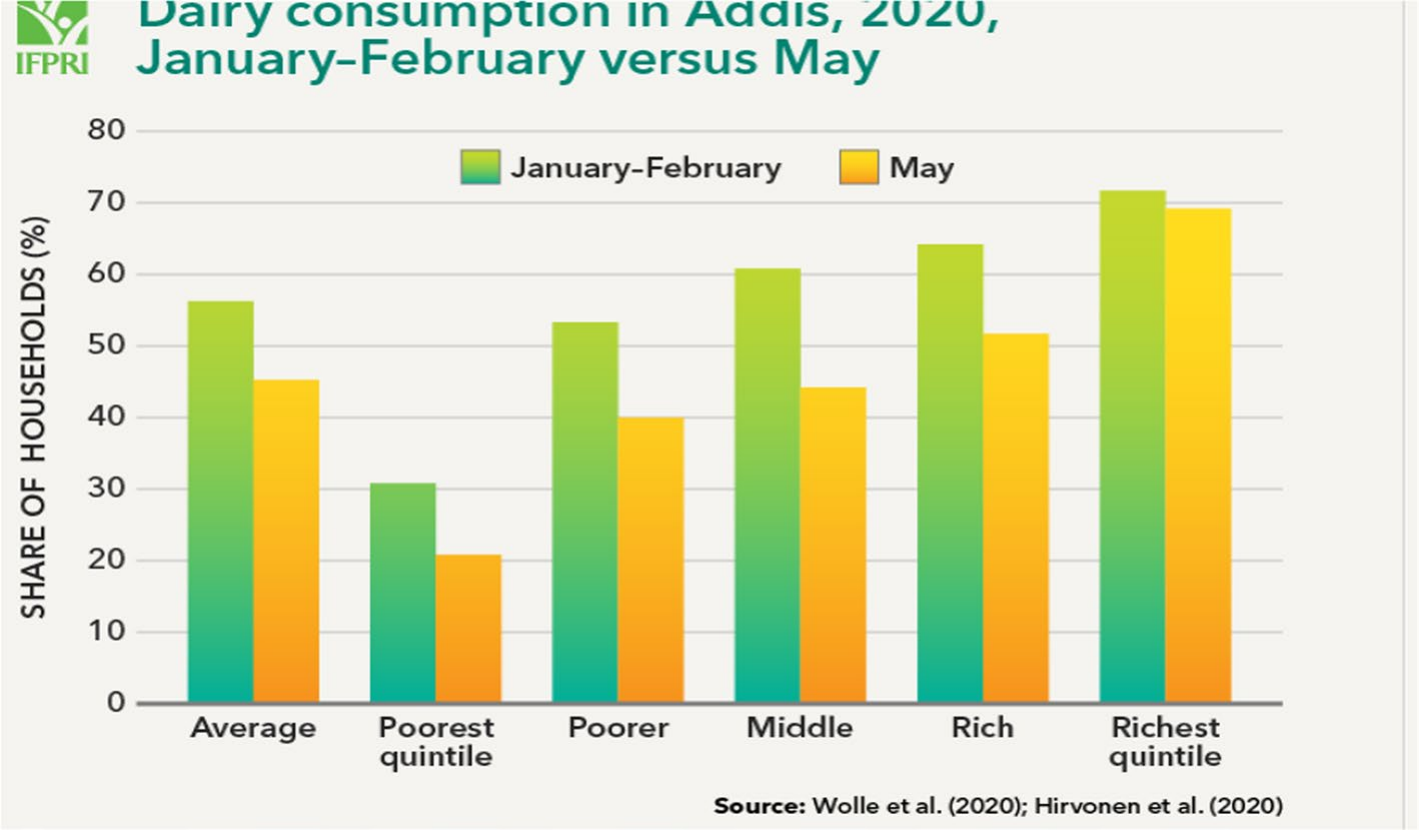
Source: As cited in Agajie et al. (2020)

Based on Agajie et al. (2020), data from two recent household surveys indicates that the consumption of dairy products in Addis Ababa has decreased since the start of the COVID-19 crisis. In Jan–Feb. 2020 56% of residents questioned and said that they consumed dairy products in the previous 7 days. In May, this number declined to 45% of interviewed households. All income groups decreased their consumption, except for the richest quintile where the share of consuming households changed little (Fig. 2).

According to Africa trade and COVID-19, 2020, Trade volumes for Africa are projected to decrease by 8% for exports and about 16% for imports for 2020, compared with previous historic trend estimates (World Trade Organization, 2020). As a result, Africa is expected to be hit particularly hard, as 17% of the world’s ‘COVID-induced’ poverty will be located on the continent, second to East Asia, the continent with the highest concentration of ‘new poor’ (20%) (Sumner, et al., 2020). Fears of a roll-back on Sustainable Development Goal achievements have been at the fore (Solber, et al., 2020), with some estimates reporting that 40–59 million more people could be pushed into extreme poverty in Africa, adding to the current 455 million people.^28^

The closure of many informal markets in the urban and peri-urban areas to avoid crowding has disrupted food supply systems, especially for fresh produce, such as meat, eggs and milk. In view of this, shifts in consumer demand have been reported. The impact is felt mostly in low-income urban households who rely on these informal food markets. Middle- and higher income families can buy fresh produce from supermarkets and grocery shops. The livelihoods of fisheries and aquaculture actors will also be negatively affected due to decreases in consumer demand, disruptions in markets access and logistical bottlenecks (FAO, 2020). For pastoralists, there will be the loss of income from selling livestock and products (milk, butter, gee and eggs), resulting in an increased reliance on the environment (charcoal burning) and overall reduction in the purchasing power of households. According to Senashaw (2020), Sales patterns various perishable products before and after the outbreak of the COVID-19. Before the outbreak the sold vegetables by the wholesalers were 19.4 and become declined to 16.7 after the pandemic. The volume of sold by consumers, micro fruit and vegetable sellers, and institutions like universities jails, army and hospitals shows big slow down since the ban measure on social distance, traveling, volume of sale and lockdown. A similar work was reported by united nation in which real-time data on food movements by commodity types is helping to reduce uncertainty and curtail any panic-driven action by countries against external or internal obstacles to trade. When milk and dairy products, fruits and vegetables, meat and fish fail to reach wholesale and retail markets, farmers, pastoralist households, fisher folks and traders suffer major income losses. This leaves fewer resources for preparing for the next season’s planting, fish catches or livestock raising and slaughter. In addition, significant amounts of food that reach retailers and consumers are wasted because of restaurant closures and hoarding by consumers who fear loss of access to retail stores (UN, 2020) (Table 1).

**Table 1:** Procurement locations and sales patterns of urban wholesalers before and after the onset of COVID-19 pandemic

	Three months before (%)	Now (%)	Differences (%-point)
Origin of vegetables			
East Shewa	44.7	60.0	15.3
Other areas	55.3	40.0	− 15.3
Clients sold to			
Other wholesalers	19.4	16.7	− 2.7
Consumers	2.3	0.0	− 2.3
Institutions (Schools, Universities, jails, hospitals, etc	6.4	1.2	− 5.2
Restaurants	6.5	11.3	4.8
Supermarkets	8.7	12.8	4.1
Micro fruit and vegetables sellers	40.5	24.8	− 15.7
Fruit and vegetable grocery shops	17.4	33.2	15.8
Other clients	0.8	0.0	− 0.8

Source: Senashaw (2020)

## COVID-19 and women entrepreneurs in Ethiopia

Women, on average, comprise 43% of the agricultural labour force in developing countries and account for an estimated two-thirds of the world’s 600 million poor livestock keepers. Of those women in the least developed countries who report being economically active, 79% report agriculture as their primary source of livelihood (48% of economically active women worldwide).^29^

The study conducted by Bashir (2021) show that the educational level, family size, region (SNNP, Gambella, Harari, Dire Dawa, and Addis Ababa), parents’ educational level, number of financially dependent people, business experiences, and access to raw materials were positive predictors of the income of women entrepreneurs. It is also found that entrepreneurial area (Afar, Amhara, and Oromiya), marital status (divorced and widowed), entrepreneurship training, enterprise’s license, and lack of supporting institutions were negatively related with the income of women entrepreneurs. Women in the urban informal sector and employed in industrial parks and the women run the risk of being among the hardest hit by the pandemic because of their specific roles in the economy and society and pre-existing, often structural, factors that have inhibited their development progress in Ethiopia. Moreover, there is a very real risk—again, if global experience and local conditions are any guide—of increased violence against women and girls (VAWG) as economic and social conditions deteriorate including increased risks of violence in lockdown and quarantine.^30^

In 2018, urban unemployment was estimated at 19.1% with youth unemployment at 25.3% where female youth takes the main share of unemployed youth (one out of three female youth in the labour market are not employed). Out of the 42% employed females, 54% are employed in the informal sector mainly in SMEs. Disaggregating using the Government of Ethiopia’s (GoE) definition of MSMEs4 and recently reported data, the country is estimated to have 800,000 micro enterprises, 12,000 small enterprises and 8000 medium-sized enterprises. Some sources suggest that the total number of MSMEs has now reached about 1.43million MSE Development Strategy.^31^ Overall, it is estimated that MSMEs employ approximately 5 million people nationwide (GoE Impact Assessment COVID-19, 2020). Worryingly, not only is the proportion of female MSE owners low, but the average monthly earnings of male production workers were reported to be 20–25% higher than their female counterparts (UNE, 2020). COVID-19 become a serious impact in addition to these mentioned women related problems and their low position and participation in economic activities with low payments.

The working age population in Ethiopia (between 15 and 64 years of age) is relatively higher than the global average, given that Ethiopia is a young country with 70% of the population below the age of 30. With an overwhelming majority (80%) of the population living in rural areas, agriculture remains the main occupational sector creating employment for 68% of the labour force in 2017. Women provide the majority of the agricultural labour force; however, their contributions often go largely unrecognized and their fathers or husbands often restrict access to resources and community participation (JCC, 2020). With an urban population of 18.7 million (53.5% of whom were female) in 201,812, urban areas have a working age population of about 15 million of whom half (7.5 million) are employed. Of these 3.9 million (52%) are estimated to be wage/paid employees that work in the government and private sectors, 3 million (41%) are self-employed and about half-a-million are ‘unpaid family workers. Based on the same survey, the unemployment rate in urban areas was estimated to be 19.1%; the corresponding rates for men and women were 12.2% and 26.4%, respectively, revealing the women are at a significant disadvantage in the urban labour market. It was higher than the average among youth between the age of 15–29 years (25.3%), pointing to another source of disadvantage—and vulnerability—in urban labour markets.^32^ Having a significant amount of disparities among women and men labor in an economy and agriculture, the pandemic is leading women in to more disadvantageous and affected groups. Other studies came with similar findings where gender inequities have also been exacerbated by the crisis, as women face additional burdens during COVID-19—as frontline health and food system workers, unpaid care work, community work, which has increased during lockdowns.^33^ Women are also at risk of an increase in domestic violence due to the recession and confinement at home when lockdown measures are in place (FAO, 2020; WHO, 2020). Another study from Zimbabwe come with similar finding which stated that a farmer in ward 35, who was also a representative of women in agriculture, reported that she lost 200 heads of cabbages and failed to timeously sell her pigs due to reduced number of customers during the COVID-19 lockdown (Muvhuringi et al., 2021).

## Conclusion and implications

This paper is intended to identify the impacts of COVID-19 on the perishable products value chain, horticultural products, its marketing, actors along the chain especially at the producers’ level, prices, value addition and processing, distribution and consumption of the products; and the impact of COVID-19 on women in Ethiopia by using the secondary empery in addition to our observation. COVID-19 laid down a significant negative impact on perishable products and its value chain. Perishable product requires a quick supply, marketing, and export. However, the outbreak of the pandemic results in deterioration and distortion of many of this products due the social distance restriction that are imposed by the government to save the life of the peoples, transportation cut off that result in the distribution impossible, in both domestic and abroad market.

Value chains of perishable products were severely impacted, in both export and local markets, due to the perishability of commodities and the disrupted domestic distribution system following the COVID-19 outbreak. Demand for more expensive and perishable products that are often consumed in hotels and restaurants has fallen sharply in the case of milk, butter and meat, and the demand for products that are believed to increase susceptibility to COVID-19—cabbage and tomato. This, in turn, leads to even more job losses along these value chains, income from remittances has declined, because the diaspora residing in other countries face income and job losses due to the measures taken and the national poverty rate rose by 9% during the lockdown period. Similarly, the consumption of the products has declined due to the fall in the volume of sold by consumers, micro fruit and vegetable sellers, and institutions like universities jails, army and hospitals shows big slow down since the ban measure on social distance, traveling, volume of sale and lockdown. From this evidence, the perishable products’ value chain is highly affected than other durable products in the era of COVID-19 which implies the need to intervene to avoid the loss and effects on small holders by facilitating marketing, distribution, processing, value addition and strengthening the linkage among the actors while save guarding the society from the pandemic.

The movement of food commodities through supply chain from surplus producing to deficit areas was constrained due to the pandemic through panic purchases, restrictions, transporters’ fear of travel and farmers withholding food for their own households. As a result, prices changed significantly over the period, with increases over a 3-month period up to 64% (tomato) and decreases as high as 67% (green pepper). Changes at the retail level were, however, relatively much smaller, between 19% increases (tomato) and 29% decreases (green pepper). Moreover, the main export market of livestock during the Ramadan and Hajji was affected and a loss of revenues from live animal exports (24.3 million kgs exported for a value of USD 45.8 million). A decrease in live animal commercialization in the country will have severe repercussions on meat and meat products (17.7 million kg valued at USD 88.6 million), and textile industry including leather and leather products (5.6 million kg valued at USD 117.4 million. This implies, it has an impact on right to food by vulnerable and marginalised groups, such as older persons, homeless female headed households and compromise their ability to meet required dietary needs and a significant reduction of foreign earnings requiring the need for intervention.

Ethiopia has lost about $25 million—almost 10% of annual revenue—just over $10 million within the horticultural sector and around 50,000 workers could lose their jobs (mostly female labourers). Regrettably, quantities of horticulture produce losses from spoilage (15%) and market glut (60%) is significant especially during the peak production periods. The produce losses are caused also by the limited access to higher value urban markets by the smallholder horticulture producers implying the effect of COVID-19 is more severe for smallholders engaged in producing horticulture if the necessary safeguard is not taken. In addition, the GDP loss in food processing was 12% in the first weeks of the outbreak. Disruptions in processing, in particular for meat, can “disconnect” supply and demand, creating simultaneous surpluses for producers and shortages for consumers, while for some specific products demand has also decreased, leading to a temporary oversupply. Therefore, the implications from the above evidence implies, the COVID-19 pandemic has placed unprecedented stresses on food supply chains, with bottlenecks in farm labour, processing, transport and logistics, as well as momentous shifts in demand from which most of these disruptions are a result of policies adopted to contain the spread of the virus due to the lack of peoples knowledge in protecting the newly outbreak of COVID-19 and the system is shifted since peoples are moving safely after the awareness creation.

Another important issue in the talk of COVID-19 impact is the issue of women as a cross-cut problem due to a high participation of women in vegetable and fruits sector, horticultural sector, livestock sector, processing industry and SMEs. Out of the 42% employed females, 54% are employed in the informal sector mainly in SMEs. Worryingly, not only is the proportion of female MSE owners low, but the average monthly earnings of male production workers were reported to be 20–25% higher than their female counterpart. COVID-19 becomes a serious impact in addition to these mentioned women related problems and their low position and participation in economic activities with low payments. Having a significant amount of disparities among women and men labor in an economy and agriculture, the pandemic is leading women in to more disadvantageous and affected groups.

## Data Availability

There is a number of data that were used to write up this article and hence, we confirm that data is available.

## Abbreviations

COVID-19: Corona virus disease 2019
FAO: Food and Agriculture Organization
FY: Fiscal year
GDP: Gross domestic product
GSC: Global supply chain
IFPRI: International Food Policy Institute
RCA: Rapid country assessment
UNCTAD: United Nations Conference on Trade and Development
USA: United State of America
USD: United State Dollar
WB: World Bank
WTO: World Trade Organization

## Footnotes

1 The detail can be obtained at http://www.fao.org/documents/card/en/c/ca8657en.

2 See the work paper prepared by Richards and Rickard (2020).

3 See Kumar Das and Bijeta Mohanty: The impact of Covid-19 on smallholder farmers in India and the way forward.

4 Take a look at https://media.ifrc.org/ifrc/press-release/east-africa-red-cross-raises-alarm-triple-menace-floods-covid-19-locusts/.

5 Assess here for more information http://www.fao.org/ag/locusts/en/info/info/index.html.

6 https://www.nature.com/articles/d41586-020-01181-3?proof=trueMay%2525252F.

7 http://www.ifpri.org/blog/ how-covid-19-may-disrupt-food-supply-chains –developing-countries.

8 See https://www.processalimentaire.com/vie-des-iaa/covid-19-la-filiere-viande-sous-tension?sso=1590405164.

9 See https://www.euractiv.com/section/agriculture-food/news/coronavirus-outbreak-exposing-vulnerabilities-in-theeu-meat-sector/.

10 UNE (2020): Proposal for Socio-Economic Response and Recovery in the Aftermath of COVID-19, ADDIS ABABA, 28 JUNE 2020.

11 See footnote 6 for more details.

12 National Agricultural Extension Intervention Programme and others using a sample focused on maize, wheat, sorghum, teff and barley.

13 http://essp.ifpri.info/2020/04/06/the-coronavirus-disease-covid-19-crisis-and-food-value-chains-in-ethiopia-insights-from-vegetable-value-chains/.

14 To understand the above table more see UNE (2020) which is written on sosio-economic impacts of COVID-19 in the country.

15 IFPRI (2020) has said a lot on this area. You can assess the information from this source to enrich your understanding on a wider scope. https://www.ifpri.org/blog/poverty-and-food-insecurity-could-grow-dramatically-covid-19-spreads.

16 For a detailed discussion of disruptions in the fruit and vegetables sector, see OECD Scheme for the Application of International Standards for Fruit and Vegetables, “Preliminary Report: Evaluation of the Impact of the Coronavirus (COVID-19) on Fruit and Vegetables Trade”, 11 May 2020, https://www.oecd.org/agriculture/fruitvegetables/oecd-covid-19-impact-on-fruit-and-vegetables-trade.pdf (TAD/CA/FVS/WD(2020)1/REV7).

17 See for example what is released by World Bank in April and June 2020.

18 IFPRI Blog, 2 April 2020, https://www.ifpri.org/blog/how-covid-19-may-disrupt-food-supply-chains-developing-countries.

19 See Rabobank, “European Food Supply: Business Is Far From Usual”, April 2020, https://research.rabobank.com/far/en/sectors/regional-food-agri/european-food-supply-business-far-from-usual.html; and Bloomberg Business, “Food Makers Get Shot of Reality Now that Panic Buying Has Waned”, 16 April 2020, https://www.bloomberg.com/news/articles/2020-04-16/with-panic-buying-waning-big-food-sees-life-without-restaurants.

20 See, for example, David Laborde, Will Martin and Rob Vos, “Poverty and food insecurity could grow dramatically as COVID-19 spreads”, 16 April 2020, IFPRI Blog, https://www.ifpri.org/blog/poverty-and-food-insecurity-could-grow-dramatically-covid-19-spreads.

21 Assess this link to obtain additional evidence https://news.trust.org/item/20200411032043-83609 and https://www.wur.nl/en/Research-Results/Research-Institutes/centre-for-developmentnnovation/show-cdi/African-horticulture-sectors-hit-hard-by-COVID-19.htm.

22 See for example this link to take advantage more information on flower dump www.ibtimes.sg/millions-flowers-dumped-floral-demandtakes-hit-due-coronavirus-outbreak-41474).

23 See for example FAO (2020).

24 See RCA (2020).

25 See IFPRI (2020).

26 Take a look at the study of Kalle Hirvonen, Belay Mohammed, Bart Minten, and Seneshaw Tamru, (2020).

27 Look at the work of Sitko and Jayne (2014), Minten et al. (2017).

28 Have some time to look at (Save the Children, 2020) if necessary.

29 See also Policy Brief on COVID-19 Impact on Women by using the link http://www.fao.org/gender/resources/infographics/the-female-face-of-farming/en/.

30 Frequently the domestic violence are increasing during the pandemic as they tend to during emergencies: www.channelnewsasia.com; www.theglobeandmail.com; www.themarshallproject.org.

31 Latest GTP I and II data for the MSMEs as compiled by Federal Urban Jobs Creation and Food Security Agency, 2020.

32 CSA, Statistical Report on the 2018 Urban Employment/Unemployment Survey, Addis Ababa, October 2018.

33 You can expand your evidence from the work of McLaren et al., 2020; Power, 2020.
